# A decade of collaboration in medicines regulation: healthcare professionals engaging with the European Medicines Agency

**DOI:** 10.3389/fmed.2024.1399947

**Published:** 2024-06-05

**Authors:** Ivana Silva, Giulia Gabrielli, Juan Garcia Burgos, Isabelle Moulon, Gonzalo Calvo Rojas, Ulrich Jaeger, Rosa Giuliani

**Affiliations:** ^1^European Medicines Agency, Amsterdam, Netherlands; ^2^Department of Clinical Pharmacology, Hospital Clinic Barcelona, Barcelona, Spain; ^3^Medizinische Universitsat Wien, Vienna, Austria; ^4^Clinical Department for Haematology and Hemostaseology, Vienna General Hospital, Vienna, Austria; ^5^Guy's Hospital, London, United Kingdom

**Keywords:** European Medicines Agency, stakeholder engagement, healthcare professionals (HCPs), public Health, regulation of medicines

## Abstract

The article shows that the input given by healthcare professionals (HCPs) adds value to the regulatory processes surrounding the development, authorisation, and monitoring of a medicine, but is also an instrument for accountability, trust, mutual exchange as well as an insight into the public health issues that matter most to one of the key stakeholder groups the Agency works with. We highlight the role of HCPs in the EU regulatory process and take stock of the first 10 years of the Framework for Interaction with HCPs to describe how practises have evolved over this time to meet the goals of informing, consulting and improving trust in the EU regulatory system. We will analyse what led European Medicines Agency (EMA) to develop this framework through to the next steps and where the interaction might lead in the future.

## 1 Introduction

Stakeholder engagement is a fundamental part of the work of the European Medicines Agency (EMA) and was coded in EU regulation from the outset of the establishment of the Agency (1).

**Figure 1:**
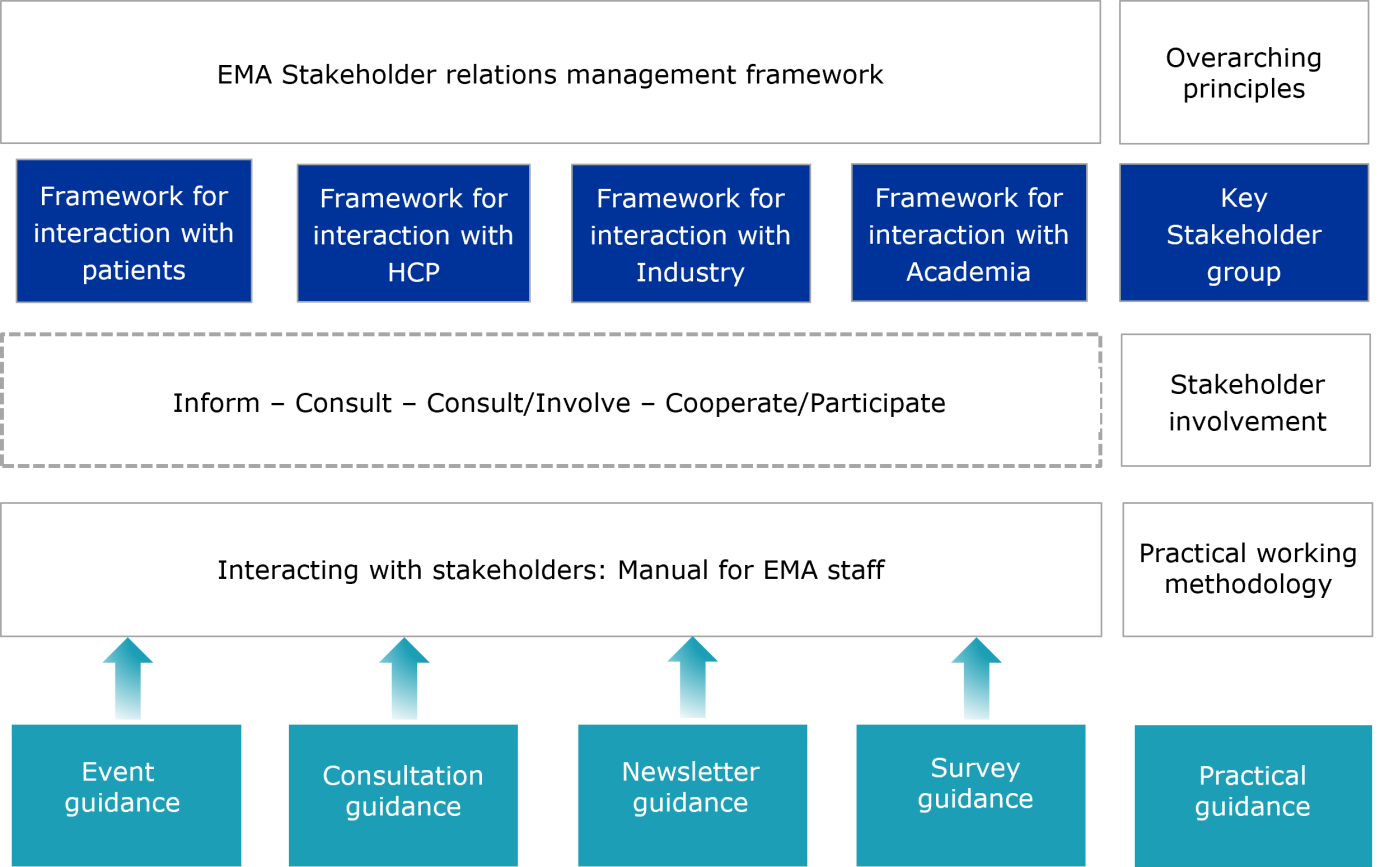
Frameworks for engagement.

European Medicines Agency’s Stakeholder Relations Management Framework (2), which was set up to give structure to its interactions with stakeholders, identifies four main groups EMA regularly engages with: patients and consumers, healthcare professionals (HCPs), academia, and industry. For each of these stakeholder groups, specific frameworks for engagement have been developed over the years (Figure 1).

The recognition of appropriate ways to engage with stakeholders in general, and HCPs in particular, is an important part of the framework of interaction to improve regulatory output, to allow knowledge exchange and to establish understanding of the Agency’s role and trust in its work.

Simply put, the work of EMA could not exist in a silo, so different ways for engagement need to aim not only at informing and communicating with HCPs, but also consulting them and their expertise, and allowing them to join the Agency’s regulatory partners and other stakeholders at the decision-making table.

## 2 Assessment of the framework

The framework for interaction with HCPs evolved over time and, whilst clearly underpinning the goals of engagement, it allows for the progressive development of new engagement methods to gather and exchange knowledge.

The first framework for interaction with HCPs was developed in 2011 (3). Before that, EMA already worked with thousands of HCPs, acting as European experts (4) in the Agency’s scientific committees, working parties and scientific assessment teams.

Most of these experts were HCPs nominated by the national competent authorities of the European Union (EU) and European Economic Area (EEA). In this capacity, HCPs were already involved as experts in the evaluation of medicines. However, the framework incorporated further ways of engaging healthcare professionals and their organisations.

Through the different activities encoded by the framework, the Agency encouraged a dialogue with HCPs with the aims of facilitating their participation in the Agency’s work in relation to medicines for human use, enhancing targeted communication and raising awareness about and trust in EMA’s activities.

After the adoption of the framework for interaction, the EMA Healthcare Professionals’ Working Party (HCPWP) was established in 2013 as a consultative body of EMA and its scientific committees (5).

The framework has evolved in the past 10 years. It was revised in 2016 to incorporate the experience of the first years of implementation, and it will be revised again as engagement activities with HCPs, and also veterinarians, and the regulatory environment continue to evolve.

## 3 Actionable recommendations

The experience gained over 10 years of implementation but also throughout the formative years of discussion and preparatory work, leads to suggest the following concrete recommendations: (1) develop and implement different levels of representation and diverse methods for engagement; (2) progress with a stepwise approach, incorporating learnings from pilots and adjusting to the specificities of the stakeholder group as well as to changing regulatory needs.

### 3.1 Levels of representation and methods for engagement

Healthcare professionals engaging with the Agency (including doctors, nurses, pharmacists, and dentists) offer a unique perspective to medicines development and regulation. In its engagement efforts, EMA strives to bring the perspectives of these different professional groups, as they are the end-users prescribing, dispensing, and administering the medicines approved, and they translate regulatory decisions into day-to-day work affecting the life of patients. These HCPs ensure that the needs and concerns of a wide range of healthcare professionals and learned societies across Europe are represented via direct contact with EMA.

They engage with EMA, from early stages of medicines development throughout the lifecycle of a medicine, policy development and regulation implementation, on three different levels of representation. These have proven to support impactful engagement in EMA activities and should be maintained.

#### 3.1.1 Level 1: HCPs representing their community

Healthcare professionals are members of the EMA Management Board and in this role represent their professional community at large.

The Management Board consists of 36 members, appointed to act in the public interest, who do not represent any government, organisation or sector. As set out in Regulation (EC) No 726/2004, one representative of doctors’ organisations, and one representative of veterinarians’ organisations are members and are appointed by the Council of the EU, following an open call and in consultation with the European Parliament (6). Board members are appointed for a 3-year term, which may be renewed.

The Board sets the Agency’s budget, approves the annual work programme and is responsible for ensuring that the Agency works effectively and co-operates successfully with partner organisations across the EU and beyond.

Healthcare professionals are also appointed as members representing their professional community in three of EMA’s scientific committees: the Pharmacovigilance Risk Assessment Committee (PRAC), the Committee for Advances Therapies (CAT), and the Paediatric Committee (PDCO). Their appointment is similar to that of HCPs sitting in the EMA Management Board. In their role as members of scientific committees, HCPs contribute to committee discussions and activities by bringing their expertise and the views of the wider community of healthcare professionals to support and reinforce existing knowledge within the European regulatory network (7).

This form of representation of HCPs is now key in all governance structures at EMA; the Emergency Task Force (ETF) (8); the Big Data Steering Group (BDSG) (9), the Darwin EU® Advisory Board (10) and both the Executive Steering Groups on Shortages and Safety of Medicinal Products (MSSG) (11) and of Medical Devices (MDSSG) (12). Today, no major initiative concerning stakeholders is initiated without considering the inclusion of a representative of HCPs.

#### 3.1.2 Level 2: HCPs representing their organisations

Healthcare professionals are members (and alternates) of the HCPWP, acting as representatives of their medical societies or professional groups, on behalf of eligible HCP organisations.

In this role, they also take part in Agency activities such as the Cancer Medicines Forum (13) and the ACT EU multi-stakeholder platform advisory group (14).

The HCPWP provides recommendations to EMA and its Human Scientific Committees on matters of direct or indirect interest to healthcare professionals in relation to medicines for human use and monitors the overall interactions between EMA and healthcare professionals. This EMA working party facilitates dialogue and exchange with healthcare professional organisations on relevant issues related to medicines and provides a forum for EMA to inform and obtain input and feedback from healthcare professionals on various EMA activities. The HCPWP also contributes to EMA’s strategic goal of advancing public health by supporting its initiatives to bringing experience from real life in the clinical setting into regulatory science and promoting a safer and more rational use of medicines. The HCPWP is consulted from the outset on major initiatives on major public health topics, for example on the introduction of biosimilars in Europe, on shortages or on antimicrobial resistance. It consists of 30 members, including 22 EU organisations and six EMA scientific committee representatives.

Healthcare professional organisations need to fulfil eligibility criteria (15) to engage more closely with EMA activities and those organisations fulfilling such criteria are referred to as ‘eligible organisations’.

They are formally listed and acknowledged as such on EMA’s website. Eligibility status offers a fast-track for interaction with EMA. They are consulted in a variety of issues, receive targeted EMA communications and frequently assist in the identification of experts for product-specific matters. They take part in yearly trainings organised by EMA, and a selection of them are members of the HCPWP.

The criteria establish that these organisations should be not-for-profit, that they should ensure sufficient representation at EU geographic level, and that they should bring the views and interests of healthcare professionals. The criteria are openly published and state verifiable measures of legitimacy, representation, accountability to members and transparency on funding sources with defined thresholds for industry funding. These were developed through the years in collaboration with the wider community of HCPs, to represent the interests of these organisations in a realistic but transparent manner.

Today, the network of EU eligible organisations comprises a wide representation of specialised organisations (e.g., clinical learned societies), but also organisations representing professional communities (e.g., doctors, pharmacists, nurses and dentists).

Groups such as general practitioners (GPs), hospital and community pharmacists and nurses are regularly involved in EMA activities and relevant organisations of representatives of these stakeholders sit in the HCPWP. More recently, an organisation representing dentists also joined the list of eligible organisations.

These organisations also have an important role in the dissemination of information, helping to relay messages from EMA through websites, newsletters, social media and conferences.

In addition to the fact that EMA is giving a voice to the HCP organisations, this also influences mutual trust and interaction and will help to inform and stimulate learned societies to actively participate in EMA strategic planning and policy making. Another highlight is the opportunity for healthcare professional representatives to collaborate with the Patients’ and Consumers’ Working Party (PCWP) in order to align efforts with EMA.

##### 3.1.2.1 Specific example: targeted engagement with GPs and family physicians

General practitioners (GPs) are the ‘entry-door’ to the healthcare system and are involved in different steps of the patients’ medication journey, from prescription to monitoring and reporting of side effects. EMA and the three European organisations representing general practitioners (GPs), family physicians and primary care professionals in Europe signed a joint statement (16) committing to strengthening interactions in 2019. The statement contained an action plan which included involving GPs and family physicians in EMA evaluations, developing relevant communication activities and exploring further collaboration with research networks in primary care, with a focus on generating real-world evidence.

#### 3.1.3 Level 3: HCPs as individual experts

When HCP experts are responding to specific requests from the Agency’s scientific committees and working parties, taking part in discussions on the development and authorisation of specific medicines, or reviewing written information on medicines prepared by the Agency, they act as individuals. EMA uses the network of existing eligible organisations as first point of contact to identify HCP experts but also maintains a stakeholder database that maps a wide number of European organisations that may be approached depending on the expertise needed.

Healthcare professional experts can also be involved in scientific advice, guidance offered by EMA to medicine developers who seek input into how to generate robust evidence on a medicine’s benefits and risks.

In these cases, they often provide additional expertise to complement that already available within the regulatory network. Their input is critical to discussions around the real-life implications of clinical study design and clinical benefit of candidate medicines.

Once a marketing authorisation application is submitted, and EMA initiates its evaluation, as well as during the safety monitoring of medicines, there are several procedures that can involve HCPs. These include early dialogue based on a request from a scientific committee, or participation in expert meetings convened by the committees to receive input on specific questions that arise during the evaluation.

These are known as scientific advisory groups and *ad-hoc* expert group meetings. These groups, gathering external experts which include healthcare professionals and patients, are asked to respond to specific questions on the potential use and value of a medicine in clinical practise.

Healthcare professionals are also involved in the review of Direct Communications to Healthcare Professionals (DHPCs) (17) and Public Health Communications. These documents are instrumental to keep HCPs informed of major decisions or events affecting the use of medicines in clinical practise.

Individual HCP experts are included in the EMA European experts’ list following a formal nomination process which includes signing a declaration of interest. Experts can only be involved in EMA’s activities once the Agency has assessed the declared interests according to its policy for the handling of competing interests (18).

##### 3.1.3.1 Specific example: HCP consultations on medicine safety

It is also possible to involve HCPs in written consultations as well as meetings and public hearings with a broad range of stakeholder representatives, depending on the procedure and matter under discussion. This was the case with two prominent cases that came to referral at the PRAC committee: the public hearing on valproate (19) in 2017 and on fluoroquinolones (20) in 2018, both considered instrumental in terms of the recommendations that were made on risk minimisation measures and the impact on the PRAC’s final opinion. Several of these methods were embedded into the regulatory procedures, through consultation with HCPs, patients and consumers, and their organisations, as well as with the approval and involvement of the scientific committees, showing that knowledge exchange needs to happen further afield, from the ideation of new regulatory solutions.

### 3.2 Stepwise approach and continuous learning

The remit of EMA is wide and has become progressively wider with the adoption of its Extended Mandate. The future revision of the pharmaceutical legislation might bring an expansion of EMA’s role, as well as acknowledge further the role of HCPs in engaging with the Agency. Nevertheless, from the beginning of its mandate, it was clear that EMA would have to develop ties to communities of professionals with expertise in diverse and specialised remits of healthcare.

**Figure 2:**
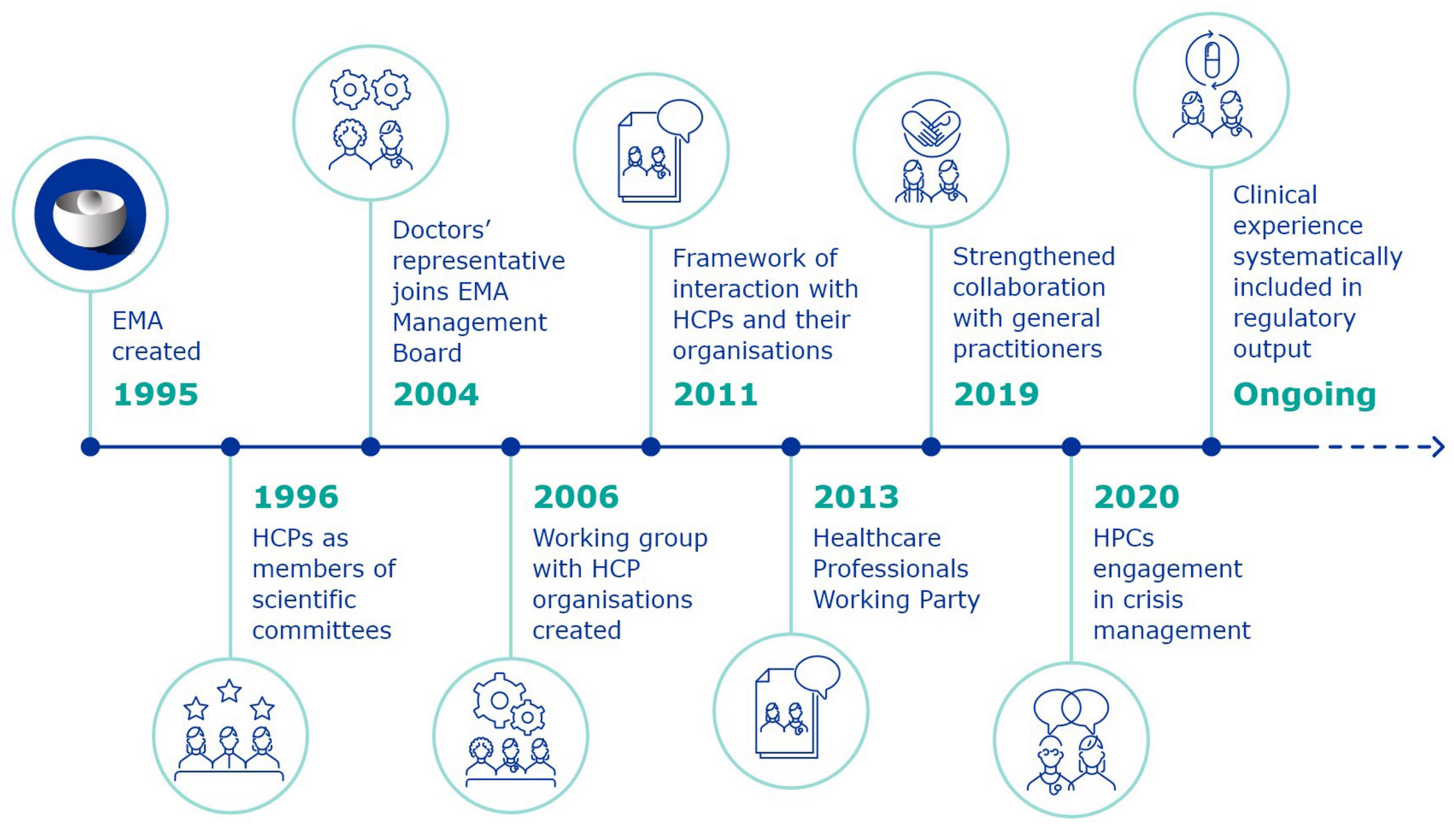
Journey of engagement with HCPs.

The framework of interaction was the result of an iterative journey of engagement with HCPs, which began in the first days of EMA (Figure 2). Following its establishment, the work of EMA with HCPs was formalised and became a structural part of the Agency’s activities. The framework recognises the different capacities of HCPs as clinical investigators, prescribers, those involved with the administration and dispensing of medicines, safety-guardians who contribute in various ways to the patient’s journey within the healthcare system. The document is not to be considered as the static result of this work, as activities and methods to enhance collaboration are constantly evolving.

European Medicines Agency was created in 1995, and beginning in 1996, individual HCP experts were nominated as members of its scientific committees, directly responsible for the evaluation and monitoring of medicines.

In 2004, a revision of the EU pharmaceutical legislation led to the inclusion, for the first time, of a representative of doctors’ organisations in the EMA Management Board. This was a significant shift, from HCPs being involved as external experts in medicines-related procedures (even as part of scientific committees), to taking a decision-making role in the Agency’s highest governance body, contributing amongst others to decisions about EMA’s budget and priorities, the appointment of the Executive Director and the monitoring of the Agency’s performance.

At the same time, a process of public consultation was launched to feed into the EMEA Roadmap to 2010 (21), which would shape the Agency’s priorities for the following years. The consultation clearly identified a need for a structured dialogue with HCP organisations, learned societies, academia and other stakeholders.

This led to the establishment, in 2005, of the first Framework for interaction with patient organisations (22), which would then be followed in 2011 by the Framework for interaction with the organisations of healthcare professionals.

The reason for this temporal lag between the two documents lies in the origins of the engagement with the two groups. Patients and consumers came to EMA and other international regulators such as the US Food and Drug Administration (FDA) following public health crises like the HIV-AIDS epidemic. They advocated to be included in a regulatory process they were not yet part of, since it impacted them so directly. HCPs already had some form of representation and inclusion in these processes, as experts and evaluators. This meant that the involvement with representatives of the counterpart HCP organisations was at an earlier stage. The interaction with patients and consumers, however, offered a good model of the added value of engaging with groups that represented communities of practitioners rather than solely with individual HCPs.

The years between 2006 and the adoption of the first framework of interaction were therefore a period of learning: consolidating a network of organisations beginning from the first workshop with HCP organisations, which led to the creation of the first informal working group of HCPs.

With the adoption of the framework of interaction, this would become, in 2013, the EMA HCPWP. Up to today, the HCPWP discusses issues of common interest between EMA and HCPs and is EMA’s main discussion forum with HCP organisations.

The HCPWP and its patient counterpart, the PCWP, both provide input and feedback into the Agency’s activities. Initially the working party discussions were procedure-related: such as the 2014 workshops on regulatory and methodological standards to improve benefit–risk evaluation of medicines and benefit–risk communication, the 2015 workshop on risk minimisation measures, and the 2016 joint session on communication and information on medicines. Soon, however, these working parties also became the Agency’s contact point on what stakeholders thought of wider topics affecting public health, with workshops being organised on themes ranging from social media (2016), antimicrobial resistance (2017), personalised medicines (2017) to use of secondary data and privacy (2020), to patient experience data (2022), EMA’s Extended Mandate (2022) and shortages (2023) (23).

As the years go by, EMA’s priorities evolve, leading to changing needs and improvement on the basis of acquired experience. The 2016 revision of the framework of interaction with HCPs (24) allowed for alignment with the European Commission’s Staff Working Document on Better Regulation Agenda (25). It also harmonised the document with EMA’s priorities expressed in the EU Medicines Agencies Network Strategy to 2020: Working together to improve health (26) to cover the full spectrum of healthcare, including primary care, and focus on the patient’s journey. This meant increasing the engagement with general practitioners which was formalised in 2019.

Throughout the years, EMA has also maintained contact with international regulatory agencies (e.g., United States, Canada, Australia and Japan) to continuously exchange experiences and learn from each other.

#### 3.2.1 Specific example: engaging with HCPs in a crisis context

In crisis management, engagement remains crucial to actively listen to the public and stakeholders and ensure their involvement in EMA activities. This includes gathering critical input into crisis-related activities in the context of the crisis; supporting specific considerations, such as discussions on therapeutics and vaccine development, associated social challenges, and combating dis-and misinformation; channelling public health messages directly to patients, healthcare professionals and citizens; and reinforcing legitimacy of actions and trust in the scientific outcomes and the EU system.

During the COVID-19 pandemic, EMA’s communication approach relied on three main pillars: communicating proactively, engaging with the public, media and healthcare professionals, and enhancing transparency on EMA’s regulatory processes and outcomes (27).

This led to increased involvement of HCPs with EMA. Soon after the announcement of the pandemic by the WHO, a COVID-19 EMA pandemic Task Force (ETF) was established where representatives from patients and HCPs were included.

The focus of the group was to facilitate discussion in order to accelerate the development and availability of treatments and vaccines for COVID-19.

Healthcare professionals were also a big part of the COVID-19 Public Meetings organised by EMA. With the expansion of EMA’s mandate in 2022, ETF was extended to provide scientific advice on the development of products intended for use during any public health emergency, reviewing scientific data, providing recommendations on the use of unauthorised medicines, and coordinating independent vaccine effectiveness and safety monitoring studies. An HCP representative continues to be part of the group.

Healthcare professionals and other stakeholders were consulted by EMA to test the usability of key information tools and visual materials for its COVID-19 vaccine outreach strategy at critical moments in the pandemic when the eyes of the public were on the Agency and its regulatory decisions (28). The increased visibility in this moment tested the level of trust of all stakeholders. Their support was key to the implementation of EMA’s decisions and, more generally, to the management of the health crisis.

## 4 Discussion

In 2021, a survey was conducted to assess the views of eligible HCP organisations on their engagement with the EMA, with the intention of establishing a baseline understanding of their perceptions in this regard. The survey showed a generally high level of satisfaction with the engagement activities with EMA.

Participation in a forum for discussion on issues of general interest, first-hand involvement in EMA activities and access to direct information were perceived as the main benefits of being an eligible organisation.

Up to 2023, the number of eligible HCP organisations has grown considerably, and the majority have identified policy officers to maintain regular contacts with EMA, facilitate active contributions in EMA activities, including consultations, and handle EMA requests for experts. This has led to the establishment of an HCP Policy Officer Group (POG) whose activity was piloted during 2021 (29) and re-assessed in 2023. The 3-year experience has shown that this is a valued group to strengthen engagement of eligible HCP organisations in EMA activities, supporting the link between EMA strategic priorities, the HCPWP workplan and the policy work developed by the organisations. It has also stimulated organisations to identify areas of further collaboration. A concrete output of such collaboration has been the publication of an article proposing a set of recommendations to improve the critical appraisal and the regulatory review of medicines taken by frail older adults (30). The HCP POG will continue its activity as a way for ‘stakeholder listening’ and enhanced early engagement with all eligible HCP organisations through policy officers.

Healthcare professionals and patient and consumers are groups that have historically worked together in their interactions with EMA, with the PCWP and HCPWP regularly meeting jointly. This close interaction will continue to be nurtured through joint discussions and, when needed, in the form of joint drafting groups convened to develop more specific areas of work. A recent example illustrating such collaboration was the PCWP/HCPWP joint submission to the public consultation on the International Council for Harmonisation (ICH) E6(R3) draft Guideline on Good Clinical Practise (GCP), in September 2023 (31).

Multistakeholder engagement, which also involves developers of new medicines and innovative treatments, like industry, SMEs and academia, is being progressively recognised as the best way to approach big systemic changes in medicine, science and technology, and the impact these will have on the work of the EU Medicines Regulatory Network. Although EMA has routinely organised multi-stakeholder discussions for key topic areas, by design the latest scientific, technological and regulatory advances require earlier involvement of all relevant parties. EMA has thus developed a more structured and systematic approach to engaging with stakeholders at a multi-stakeholder level on strategic areas concerning both product-related activities and public health priorities. Early engagement with all stakeholders to understand each other’s perspectives, promote dialogue and facilitate cooperation has been a recurring theme that will continue to be promoted as much as possible in the future. In 2022–2023, for example, multi-stakeholder engagement targeted priority areas such as crisis management and preparedness, ensuring availability of medicines and medical devices, progressing in regulatory science areas such as patient experience data, promotion of research, innovation and development.

The EU Medicines Agencies Network Strategy to 2025 outlines priority focus areas for which increased collaboration and engagement with stakeholders, including healthcare professionals, and downstream decision makers such as HTA bodies and payers will be critical to support its implementation. This is particularly relevant when explaining, preparing for, resourcing, and managing a shift to more post-licencing evidence generation as the regulatory system evolves (32).

The next years will bring new developments, opportunities but also challenges, in the field of public health. Challenges and advances in biomedical sciences and in health care systems will require increased dialogue with HCPs. Recent legislation, such as the Clinical Trials Regulation, the Health Technology Assessment Regulation, EMA’s extended mandate and legal proposals for the new pharmaceutical legislation, and European Health Data Space will lead to an increased need for dialogue with HCPs and other stakeholders. This will be essential to ensure legislation is implemented in a way which supports the needs of patients and HCPs, and that it overall improves public health.

Digital healthcare is creating new ways of treating patients, of gathering data on effects of medicines either favourable or unfavourable, of using tools like artificial intelligence to capture, use and analyse such data.

Innovation will continue in fields such as personalised medicines, cell and gene therapies and medical devices.

New expertise will have to be developed and incorporated in the network, and a lot of this expertise will come from HCPs, including younger generations of medical professionals and even current biomedical science students.

The evolution of the Framework of interaction is not over: new revisions will be necessary, guided by the increasing engagement with this essential stakeholder group.

## Author contributions

IS: Conceptualization, Methodology, Project administration, Supervision, Visualization, Writing – original draft, Writing – review & editing. GG: Conceptualization, Data curation, Investigation, Methodology, Resources, Writing – original draft, Writing – review & editing. JG: Validation, Writing – original draft, Writing – review & editing, Conceptualization. IM: Validation, Writing – original draft, Writing – review & editing, Conceptualization. GC: Writing – original draft, Writing – review & editing, Conceptualization. UJ: Writing – original draft, Writing – review & editing, Conceptualization. RG: Writing – original draft, Writing – review & editing, Conceptualization.
